# Genotyping and Zoonotic Potential of *Enterocytozoon bieneusi* in Stray Dogs Sheltered from Shanghai, China

**DOI:** 10.3390/ani11123571

**Published:** 2021-12-16

**Authors:** Hua Liu, Jie Xu, Yujuan Shen, Jianping Cao, Jianhai Yin

**Affiliations:** National Institute of Parasitic Diseases, Chinese Center for Disease Control and Prevention (Chinese Center for Tropical Diseases Research), NHC Key Laboratory of Parasite and Vector Biology, WHO Collaborating Center for Tropical Diseases, National Center for International Research on Tropical Diseases, Shanghai 200025, China; liuhua@nipd.chinacdc.cn (H.L.); xu_jie0214@163.com (J.X.); shenyj@nipd.chinacdc.cn (Y.S.); caojp@chinacdc.cn (J.C.)

**Keywords:** microsporidia, *Enterocytozoon bieneusi*, stray dogs, genotype

## Abstract

**Simple Summary:**

*Enterocytozoon bieneusi* is the most prevalent species with zoonotic risks in humans and various livestock, wildlife and companion mammals. Dogs being the most popular companion animals of humans become more and more regarded recently. The present study reported the total *E. bieneusi* positive rate of 8.8% (24/272), and 8 genotypes including three known (genotypes EbpA, Henan V and Type IV) and 5 novel genotypes (genotypes SHZJD1–5) in stray dogs from Shanghai but sheltered in Zhenjiang, Jiangsu Province, China. In addition, all the genotypes here were all clustered into group 1 with zoonotic potential.

**Abstract:**

Microsporidia are considered to be highly diverged and specialized parasites, and can infect a wide variety of vertebrate and invertebrate hosts. *Enterocytozoon bieneusi* is the most prevalent species in humans and various livestock, wildlife, and companion mammals. Dogs being the most popular companion animals of humans become more and more regarded. In this study, 272 fecal specimens were collected from stray dogs from Shanghai, but the dogs were adopted in a shelter in Zhenjiang, Jiangsu Province, China. *E. bieneusi* was examined by PCR amplification of the internal transcribed spacer (ITS) region and sequence analysis. The total positive rate of *E. bieneusi* was 8.8% (24/272). Moreover, 8 genotypes were found, including three known (genotypes EbpA, Henan V and Type IV) and 5 novel genotypes (genotypes SHZJD1–5). Two samples were positive for two genotypes, one was positive fortype SHZJD4 and Henan V, the other was positive for Henan V and Type IV. In addition, phylogenetic analysis showed all genotypes obtained in this study were all clustered into the zoonotic group 1. Therefore, the risk of zoonotic transmission of pathogens such as *E. bieneusi* from stray dogs to humans potentially threaten human health, and it is time to strengthen their health management.

## 1. Introduction

Microsporidia are obligate eukaryotic intracellular pathogens, including more than 200 microsporidian genera and nearly 1500 species. Aproximately 17 species have been reported in mammals including humans, among which *Enterocytozoon bieneusi* (*E. bieneusi*) was the most common in humans and wild, domesticand companion mammals worldwide [1,2,3,4]. Infection with *E. bieneusi* could cause chronic diarrhea and wasting syndrome in immunocompromised patients, such as HIV/AIDS patients and organ transplant recipients [5,6]. In immunocompetent individuals, it is mainly associated with self-limited diarrhea and malabsorption [4,5,7].

Recently, pets especially dogs or cats, have been a part of modern life with the increasing consumption capacity [8]. Meanwhile, more and more stray dogs/cats unfortunately appeared in cities or countries. These animals have been considered to be potential conserved hosts of various pathogens especially intestinal parasites [9]. Now, it has been reported that *E. bieneusi* may be transmitted between humans and animals through contaminated fomites, water and food [10,11].

Recently, molecular analysis combined with genotyping have been considered to be a standard tool to genetically characterize *E. bieneusi*. Nested PCR amplification and sequence analysis of the internal transcribed spacer (ITS) of the rRNA gene have been widely used in identifying and describing the genotypes of *E. bieneusi* [12]. So far, at least 500 genotypes of *E. bieneusi* have been defined based on ITS gene, which were classified into 11 phylogenetic groups. Group 1 and group 2 cover the most of zoonotic genotypes, whereas groups 3–11 seem to have strong host specificity [13].

In view of dogs are the most important companion animals of humans, and more and more stray dogs appear in the society, the present study aimed to describe the prevalence and genotypes of *E. bieneusi* through molecular characterization in adopted stray dogs from Shanghai, China.

## 2. Materials and Methods

### 2.1. Sources and Collection of Specimens

A total of 272 fecal samples from post-weaning dogs (264 *Canis lupus familiaris*, two huskies, two golden retrievers, two samoyeds, one wolf dog and one bulldog) were obtained in a shelter in Zhenjiang, Jiangsu Province, China, while the stray dogs were all transferred from Shanghai. Samples were collected individually from each dog using a sterile glove and placed in a faeces container, and then were put into a box with ice. All the specimens were sent to the laboratory and stored at −20 °C until use.

### 2.2. DNA Extraction and PCR Amplification

QIAamp DNA Stool Mini Kit (QIAGEN, Hilden, Germany) was used to extract genomic DNA according to the manufacturer’s instructions. The extracted DNA was stored at −30 °C until used in the PCR amplification. The presence of *E. bieneusi* DNA was confirmed by amplifying of an approximately 390 bp fragment including the internal transcribed spacer (ITS) region of the rRNA gene. The primers and PCR thermal cycler parameters used in the present study have been described in our previous study [5]. Briefly, PCR mixtures (25 μL) were composed of 12.5 μL of Taq mix (Promega, Madison, WI, USA), 1 μL each of the forward and reverse primers (10 μM) (Sunny Biotechnology, Shanghai, China), 1 μL of DNA template and 9.5 μL of nuclease-free water (Promega, Madison, WI, USA). The positive control (*E. bieneusi*-positive DNA) and negative control (nuclease-free water) samples were added in each PCR run. All the samples were conducted by PCR for three times to make sure the authenticity of the results. The PCR products were examined by 2% agarose gel electrophoresis with GelRed staining (Biotium Inc., Hayward, CA, USA) and observed using a gel imaging system.

### 2.3. Sequencing and Molecular Analysis

All the positive secondary amplification products were sequenced in both directions on an ABI 3730 DNA Analyzer (Applied Biosystems, Foster City, CA, USA) using a Big Dye Terminator v3.1 Cycle Sequencing Kit (Applied Biosystems) at Sunny Biotechnology, Shanghai. The obtained sequences were assembled using contigexpress. The genotypes of *E. bieneusi* were confirmed by aligning with the published sequences in GenBank and analysed using Clustal X 1.83 (https://clustalx.software.informer.com/, accessed on 11 October 2021).

### 2.4. Phylogenetic Analysis

A phylogenetic tree was constructed based on the ITS region sequences using the neighbour-joining (NJ) method in the software MEGA 7.0 (https://www.megasoftware.net/home, accessed on 8 October 2021). The representative nucleotide sequences of different *E. bieneusi* groups were downloaded from GenBank. Bootstrap analysis was used to assess the robustness of clusters using 1000 replicates.

### 2.5. Nucleotide Sequence Accession Numbers

The obtained new nucleotide sequences from this study were deposited in GenBank under accession numbers OK481084-OK481088.

## 3. Results

### 3.1. Occurrence of E. bieneusi in Stray Dogs

In the present study, 24 (8.8%) in 272 fecal samples from dogs (one samoyed and 23 *Canis lupus familiaris*) were positive for *E. bieneusi* by PCR amplification of the ITS regions. Furthermore, 12 female and 12 male dogs were identified to be *E. bieneusi*-positive among 133 female and 139 male dogs.

### 3.2. Genetic Characterizations of E. bieneusi in Stray Dogs

Of the 24 *E*. *bieneusi*-positive specimens, 8 genotypes were detected including three known genotypes (EbpA, Henan V and Type IV) and 5 novel genotypes (genotypes SHZJD1-SHZJD5). The most prevalent genotype was Type IV (41.7%, 10/24), followed by SHZJD1 (25.0%, 6/24) and EbpA (12.5%, 3/24), genotypes SHZJD2-SHZJD4 identified in one sample each. Moreover, the remaining two samples were detected to be positive for two genotypes, one was positive for genotypesSHZJD4 and Henan V, and the other was positive for Henan V and Type IV (Table 1). In addition, the sequences of genotypes SHZJD2-SHZJD4 have the largest similarities while genotype SHZJD1 was obviously different from them based on sequence analysis (Table 2).

### 3.3. Phylogenetic Analysis

**Figure 1:**
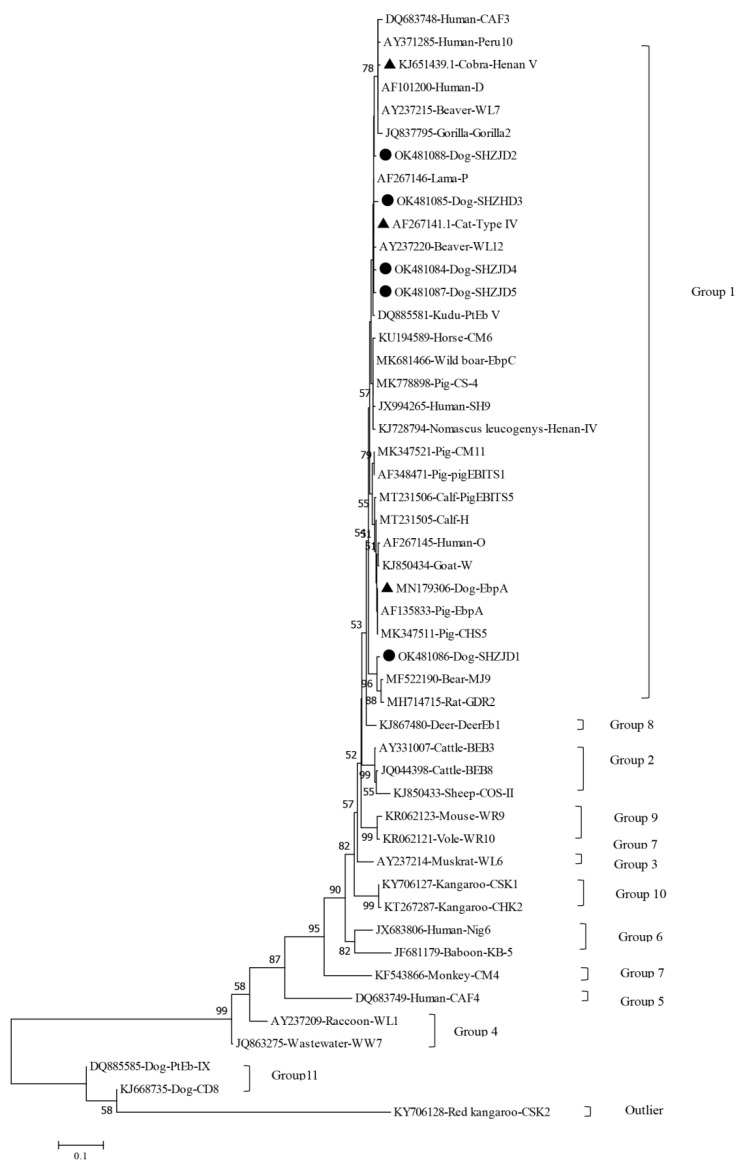
Phylogenetic relationships of the 5 novel genotypes (SHZJD1 to SHZJD5) and three known genotypes (EbpA, Henan V and Type IV) of *E. bieneusi* identified in this study inferred from a neighbour-joining analysis of internal transcribed spacer (ITS) gene sequences based on genetic distances calculated by the Kimura two-parameter model. Numbers on the branches are percent bootstrapping values from 1000 replicates. The *E. bieneusi* genotype CSK2 (KY706128) from a white kangaroo was used as the outgroup. The black filled-in triangles and circles indicate novel and known genotypes identified in this study, respectively.

Phylogenetic and nucleotide sequence analysis showed that all genotypes including the novel genotypes (SHZJD1-SHZJD5) and the known genotypes (EbpA, Type IV and Henan V) obtained in this study were clustered into group 1 (Figure 1), indicating the stray dogs posed potentially zoonotic transmission of *E. bieneusi*.

## 4. Discussion

The study presented the positive percentage of *E. bieneusi* in stray dogs sheltered from Shanghai by nested PCR amplification based on the ITS regions, and a total of 24 samples (8.8%, 24/272) were identified to be positive for *E. bieneusi*, which is similar to the reported infection rate in Spain (8.7%) [14] and Switzerland (8.3%) [15]. In view of the molecular investigations of *E. bieneusi* infection in stray dogs worldwide, the infection rate varied from 0 to 20.5% [3,16,17,18,19]. Moreover, farm dogs were the most reported on the surveys of this parasite infection in China [20,21,22,23,24,25] followed by pet dogs [3,25,26], with the highest infection rate of 40.74% in Hebei and 11.7% in Henan respectively. In other countries, the most *E. bieneusi* infection investigations of dogs were household dogs, with infection rate from 0.8% to 19.2% [15,26,27,28,29,30,31]. The *E. bieneusi* infection of pet dogs was from 4.4% to 11.8% [32,33,34,35], and that in undefined dogs have reported to be 43.8% [1], which is higher than other sources of dogs. In addition, dogs from clinics have also been detected *E. bieneusi* infection [23,36] (Table 3). It was speculated that the differences in infection rate in dogs may be potentially attributed to the different exposure probability to the potential pathogens in various living conditions, feeding modes, geographical distributions or other factors [13,37,38].

Of the 24 *E*. *bieneusi*-positive specimens, 8 genotypes were detected, including three known genotypes (EbpA, Henan V and Type IV) and 5 novel genotypes (SHZJD1-SHZJD5). Genotype Type IV was the dominant genotype which was found in 41.7% (10/24) of *E. bieneusi* isolates. Type IV was widely distributed in both humans and animals. The second one was the novel genotype SHZJD1 with 6 samples. To the best of our knowledge, genotype HenanV was first found in dogs, and in fact it has been found in humans [39] and monkeys [40]. The presence of common zoonotic genotypes EbpA and Type IV were found dogs, which is consistent with previous studies in Colombia [41], Henan [40], Shandong [24] and Xinjiang [25,42] (Table 3). Interestingly, two samples were positive for two genotypes in the study based on all of the genes being amplified at least three times and all PCR-positive products were sequenced successfully in both directions for accurate analysis, with one being positive for type SHZJD4 and Henan V, the other was positive for Henan V and Type IV. To our knowledge, there was only one previous report on coinfection of this parasite [25]. In addition, most stray dogs live in various complicated environment might contribute to coinfection. It has been reported that coinfection of different genotypes is prone to mutation or genetic recombination which may pose a greater threat to public health [43].

In recent years, companion animals particularly dogs play an important role in the family life, so the opportunities for intimate contact between dogs and humans continue to increase, which is accompanied by an increasing risk of exposure to certain diseases from dogs to humans. In fact, the five cities of Beijing, Shanghai, Chongqing, Wuhan and Guangzhou have been recognized as ‘Pet City’ [8] and it was estimated that there were at least 74 million pets at the end of 2018 and there were a lot of stray dogs in China [43]. According to the World Health Organization, there were nearly 200 million stray dogs worldwide [9]. In China, it was estimated that there were about 40 million stray dogs in China by the end of 2019 (https://www.sohu.com/a/362578244_100060664, accessed on 11 November 2021). And dogs have been reported to be associated with more than 60 zoonotic diseases, such as the ones caused by protozoa, helminths and arthropods, which are dangerous to public health [8,9,44,45]. Stray dogs are also considered to be a risk factor for many diseases such as rabies or echinococcus [46,47]. The feces from stray dogs might also pose threat to water or soil resources or the surroundings. Ingestion of parasitic stages through contaminated water or food is the most important transmission route for human infection. Contact with soil is also an important route for human especially children because they are often exposure to contaminated soil while playing in sandpits or playgrounds in the park [45]. In addition, stray dogs often keep in close contact with pet dogs or other companion animals, which act as another potential infection route [48]. Now, reports about the illegal centralized transportation of stray animals including dogs are increasing, which is unfavourable for the management of animals, and there might be potential disease transmission during the cross-regional transportation [38]. Furthermore, some animal protectionists occasionally adopt stray animals as pets, which also increase the potential risk of exposure to zoonotic diseases.

Stray dogs or sheltered dogs are not well protected against parasites, it may be attributed to lack of inadequate education for people or sufficient economic resources for dogs. The living environment of stray dogs may favor the zoonotic parasites transmission [9]. The observation of *E. bieneusi* infection in stray dogs give us important warning that dog food should be used to feed dogs instead of uncooked meat or fish and regular deworming is important. Better management and control capabilities should be taken to prevent the human-animal transmission caused by pathogens carried by pets, such as people should not keep in close contact with pets, or pets should not be allowed to enter public green spaces or the riverside. What’s more, the feces of dogs should be cleaned timely and correctly especially in public places.

Here, we described the genetic characteristics of *E. bieneusi* in fecal DNA samples from stray dogs using nested PCR-based sequencing of the ITS region. Zoonotic *E. bieneusi* genotypes were identified in this study but the transmission route was poorly understood. However, the contamination resulted from stray dogs with parasitic infection is a growing public health concern. Therefore, molecular investigations should be conducted in the future surveys.

There are some limitations in the present study. First, stool samples were collected after excretion, rather than collected directly from the rectum of each dog, it may be contaminated with each other due to space sharing in the shelter. Second, this is a cross-sectional survey on the *E. bieneusi* infection of stray dogs from Shanghai but sheltered in Zhenjiang, Jiangsu, no more informaiton on the original locations of dogs was provided, and no overall number of stray dogs in the region, making it difficult to assess their representativeness generally.

## 5. Conclusions

In conclusion, we detected the presence of *E. bieneusi* with positive rate of 8.8% in stray dogs in China. Based on PCR amplification of TIS gene, 5 novel genotypes (SHZJD1 to SHZJD5) and three known genotypes (EbpA, Henan V and Type IV) were identified with Type IV being the predominant genotype, exhibiting potential zoonotic transmission. Phylogenetic analysis showed that all the genotypes belonged to group 1. For public health, further investigation on the transmission dynamics of human-companion animals and the potential transmission risk during transportation should be required. In addition, better health management needs to be taken to assure dogs’ health and prevent the potential threats to public health.

## Figures and Tables

**Table 1:** Prevalence and genotype distribution of *E. bieneusi* isolates of stray dogs in Shanghai.

Infection	Genotypes	No. of Positive
Known	EbpA	3
	Type IV	10
Novel	SHZJD1	6
	SHZJD2	1
	SHZJD3	1
	SHZJD4	1
Mixed	HenanV + SHZJD4	1
	HenanV + Type IV	1

**Table 2:** Variation of the ITS gene sequences of *E. bieneusi* isolates in dogs.

Genotype	Nucleotide at Position (ITS)																		Genbank No.
	137	187	193	199	201	207	219	223	237	239	241	244	245	250	264	268	271	282	
Novel																			
SHZJD1	A	T	T	T	G	A	T	T	A	G	G	A	A	A	T	A	T	G	OK481086
SHZJD2	G	C	T	T	G	A	C	G	G	G	G	G	G	A	T	A	G	A	OK481088
SHZJD3	G	C	T	T	G	A	C	G	G	G	G	G	A	A	C	G	G	A	OK481085
SHZJD4	G	C	T	T	G	G	C	G	G	G	G	G	A	A	T	A	G	A	OK481084
SHZJD5	G	C	C	T	G	A	C	G	G	G	G	G	A	A	T	A	G	A	OK481087
Known																			
EbpA	A	T	T	T	T	A	T	T	G	G	G	G	A	G	T	A	G	A	MN179306
Type IV	G	C	T	T	G	A	C	G	G	G	G	G	A	A	T	A	G	A	AF267141
HenanV	G	C	T	C	G	A	C	G	G	A	A	G	A	A	T	A	G	A	KJ651439

**Table 3:** Prevalence and genotype distribution of *Enterocytozoon bieneusi* in dogs worldwide.

County/Province	Source; Infection Rate	Genotype (No.)	Reference
Colombia	S, 15.0 (18/120)	1: Type IV (1), WL11 (1); 11: PtEb IX (16)	[17]
Poland	H, 4.9 (4/82)	1: D (2); 11: PtEb IX (2)	[31]
Spain	H, 19.2 (14/73)	1: A (7)	[29]
Spain	H, 0.8 (2/237)	2: BEB6 (1); 11: PtEb IX (1)	[32]
Spain	H, 11.8 (2/17)	N/A	[28]
Spain	H, 8.7 (4/46)	N/A	[14]
Switzerland	F, 8.3 (3/36)	11: PtEb IX (3)	[15]
Australia	H, 4.4 (15/342)	1: D (5); 3: VIC_dog1 (1); 11: PtEb IX (9)	[33]
Iran	S, 5.3 (4/75)	1: D (4)	[16]
Iran	P, 11.8 (2/17)	N/A	[35]
Iran	C, 25.8 (8/100)	N/A	[40]
Egypt	H, 13 (14/108)	N/A	[30]
Japan	P, 5.0 (1/20); S, 1.7 (1/59)	11: PtEb IX (2)	[18]
Japan	P, 4.4 (26/597)	11: PtEb IX(26)	[36]
Korea	U, 43.8 (21/48)	1: D(8); Korea-WL1(8); Korea-WL1(6); Korea-WL1(1)	[1]
China	-	-	-
Jilin	P, 7.7 (2/26)	2: CHN5 (1), CHN6 (1)	[44]
Henan	P, 11.7 (23/197); S, 20.5 (31/151)	1: O (4), D (3), EbpA (2), macaque3 (2), CD1-CD4, EbpC, Peru8, PigEBITS5 and Type IV (1 each); 2: CD6 (1); 7: CD5 (1); 11: PtEb IX (26), CD8 (4), CD7 (2), WW8 (1)	[39]
Heilongjiang	P, 7.2 (18/249); S, 0.0 (0/18)	1: D (1), EbpC (2), NED1 (1), NED2 (1); 11: PtEb IX (14), NED3 (1), NED4 (1)	[19]
Shanghai	H, 7.8 (8/102); P, 5.5 (21/383)	1: D (1); 11: PtEb IX (28)	[22]
Anhui	C, 9.3(20/215)	1: PtEb IX(12),EbpC(4),CHD1(2),CHD2 (2)	[23]
Zhejiang	C, 7(7/100)	1: PtEb IX(4), CHD 3(3)	[19]
Shandong	F, 6.5 (23/356)	1: D(8), Peru8(3), Type IV(11); 2:CHG1(1)	[24]
Heilongjiang	F, 4.1 (2/49)	1: D (1), CHN-R1 (1)	[20]
Heilongjiang	F, 10.5 (17/162)	1: D (14), CHN-DC1 (1), and CHN-DC1/WildBoar3 (1)	[21]
Heilongjiang	F, 2.5 (1/40)	CHN-DC1 (1)	[22]
Hebei	F, 40.74(22/54)	1: CHN-DC1 (1), CHN–F1 (3), NCF2 (15), NCR1 (2)	[20]
Jilin	F, 30.91 (34/110)	1: CHN-DC1 (7), CHN–F1 (6), D (5), NCF2 (11), NCR2 (5)	[20]
Liaoning	F, 15.28 (11/72)	1: CHN–F1 (1), D (4), NCF2 (6)	[20]
Xinjiang	H, 2.6(1/39)	1: EbpA(1)	[43]
Xinjiang	P, 6.3(38/604)	11: PtEb IX (19); 1: EbpC (12), D (2), CD9 (1), Type IV (1), CD11 (1), CD12 (1), CD13 (1)	[27]
Shanghai	S, 8.8(24/272)	1: TypeIV(10); EbpA(3); SHZJD1(6); SHZJD2(1); SHZJD3(1); SHZJD5(1); SHZJD4 + HenanV (1); HenanV + Type IV (1)	This study

Note: The data of this study are updated on the basis of the previous literature. a C, clinic; F, farm; H, household; P, pet; S, stray; U, undefined; Z, zoo; b Genotyping study using confirmed *E. bieneusi*-positive isolates.; c N/A, not available.

## Data Availability

The sequences that supports the finding of this study is openly available in the GenBank database at https://www.ncbi.nlm.nih.gov/nucleotide/ (accessed on 13 October 2021) under accession number OK481084-OK481088.
